# Reduced aboveground tree growth associated with higher arbuscular mycorrhizal fungal diversity in tropical forest restoration

**DOI:** 10.1002/ece3.2487

**Published:** 2016-09-21

**Authors:** Ellen K. Holste, Karen D. Holl, Rakan A. Zahawi, Richard K. Kobe

**Affiliations:** ^1^ Department of Forestry Michigan State University East Lansing MI USA; ^2^ Environmental Studies Department University of California Santa Cruz CA USA; ^3^ Las Cruces Biological Station Organization for Tropical Studies San Vito Costa Rica

**Keywords:** forest recovery, land use history, mycorrhizae, plant–soil interactions, productivity–diversity relationship, spore production

## Abstract

Establishing diverse mycorrhizal fungal communities is considered important for forest recovery, yet mycorrhizae may have complex effects on tree growth depending on the composition of fungal species present. In an effort to understand the role of mycorrhizal fungi community in forest restoration in southern Costa Rica, we sampled the arbuscular mycorrhizal fungal (AMF) community across eight sites that were planted with the same species (*Inga edulis, Erythrina poeppigiana, Terminalia amazonia,* and *Vochysia guatemalensis*) but varied twofold to fourfold in overall tree growth rates. The AMF community was measured in multiple ways: as percent colonization of host tree roots, by DNA isolation of the fungal species associated with the roots, and through spore density, volume, and identity in both the wet and dry seasons. Consistent with prior tropical restoration research, the majority of fungal species belonged to the genus *Glomus* and genus *Acaulospora*, accounting for more than half of the species and relative abundance found on trees roots and over 95% of spore density across all sites. Greater AMF diversity correlated with lower soil organic matter, carbon, and nitrogen concentrations and longer durations of prior pasture use across sites. Contrary to previous literature findings, AMF species diversity and spore densities were inversely related to tree growth, which may have arisen from trees facultatively increasing their associations with AMF in lower soil fertility sites. Changes to AMF community composition also may have led to variation in disturbance susceptibility, host tree nutrient acquisition, and tree growth. These results highlight the potential importance of fungal–tree–soil interactions in forest recovery and suggest that fungal community dynamics could have important implications for tree growth in disturbed soils.

## 1 Introduction

Incorporating mycorrhizal fungi into tropical restoration efforts requires an understanding of multiple ecological processes relating belowground organisms, aboveground plant performance, and site‐specific environmental variables (Heneghan et al., 2008). Mycorrhizal fungal–plant symbioses are important for maintaining soil aggregation (Rillig, 2004), increasing nutrient cycling (Read & Perez‐Moreno, 2003), and most importantly to reforestation efforts, for improving plant growth and survival (Janos, 1980). Globally, approximately 80% of plant species form symbiotic relationships with mycorrhizal fungi, and in many tropical forests, plants predominantly form arbuscular mycorrhizal fungal (AMF) associations. Despite their ubiquity and importance to ecosystem structure and function, surprisingly little is known about the abundance and diversity of AMF in tropical soils (Alexander & Selosse, 2009), and even less about their role in ecological restoration (Kardol & Wardle, 2010).

The impact of changing from a high diversity, forest habitat to a low diversity, graminoid pasture or mono‐dominant cropland can have profound effects on mycorrhizal fungal community diversity and composition (Aldrich‐Wolfe, 2007; Mueller et al., 2014; Sturmer & Siqueira, 2011). Moreover, decreases in soil nutrient availability due to land use conversions may increase AMF root inoculation as plants become more dependent on their fungal symbionts for nutrient acquisition (Smith & Read, 2008).

Whereas it is commonly assumed that a diverse mycorrhizal fungal community will enhance tree growth in forest restoration, actual AMF effects could be more complex. Fungal species richness can associate with both increases (van der Heijden et al., 1998; Vogelsang, Reynolds, & Bever, 2006) and decreases in plant productivity (Hiiesalu et al., 2014). More phylogenetically diverse AMF are likely to be functionally different (Maherali & Klironomos, 2012), and functional diversity among fungal taxa could yield distinctive impacts on plant growth (Klironomos, 2003). For example, AMF families may improve plant growth differently by either providing protection against fungal pathogens (Glomeraceae) or enhancing plant phosphorus (P) uptake (Gigasporaceae) (Maherali & Klironomos, 2007). AMF species also may vary in their tolerance to their environment and susceptibility to disturbance which can affect their relationship with plants (Jasper, Abbott, & Robson, 1991).

Plant growth is likely affected more by AMF composition than by diversity, as particular AMF‐plant associations appear to have a greater impact on the growth of specific plant species than others (Klironomos, 2003). Differences in benefits are generally associated with life history strategies in AMF species via the amount of carbon (C) extracted from their hosts (Olsson, Rahm, & Aliasgharzad, 2010), their ability to acquire nutrients (Smith, Jakobsen, & Smith, 2000), and fungal nutrient storage capacity (Kiers et al., 2011). Bever, Richardson, Lawrence, Holmes, and Watson (2009) found that host plants can preferentially allocate photosynthates to more beneficial fungal partners, and thus may “choose” symbionts that increase their growth, but only if the symbiotic fungi are spatially separated within root systems (Friese & Koske, 1991) or patches of soil (Wolfe, Mummey, Rillig, & Klironomos, 2007). Hence, changes in AMF diversity and abundance due to land use conversion may have profound effects on plant growth and restoration efforts.

**Figure 1:**
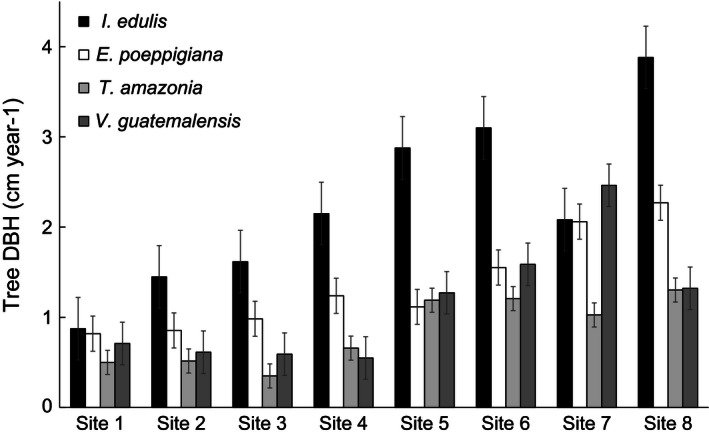
Mean annual tree diameter at breast height (DBH) growth grouped by species and site (±1 SE)

We examined the relationships between AMF community abundance and diversity and the growth of four tree species in eight‐five‐ to seven‐year‐old reforested sites in southern Costa Rica (Table 1, Figure 1). Previous research tested past land use, soil nutrients, soil compaction, and understory cover as potential causes for differences in tree growth, but only the ranked length of pasture use explained a significant amount of variation (Holl & Zahawi, 2014; Holl, Zahawi, Cole, Ostertag, & Cordell, 2011). As past land use intensity can strongly affect soil microbial communities (Carpenter, Mayorga, Quintero, & Schroeder, 2001; Oehl et al., 2010), we investigated whether differences in mycorrhizal fungal communities could help explain the influence of prior pasture use on tree growth. AMF abundance and diversity were characterized in three ways: percent colonization of host tree roots; identification of the fungal species associated with trees roots through DNA isolation; and spore density, volume, and identity in both the wet and dry seasons. Although soil nutrients explained little of the variation in tree growth, nutrient availability can alter AMF abundance and diversity (Camenzind et al., 2014; Lekberg, Koide, Rohr, Aldrich‐Wolfe, & Morton, 2007); thus, data on soil attributes were collected to better evaluate the mechanisms underlying the site, tree, and fungal differences. Specifically, we hypothesized that: *H1*) AMF abundance (i.e., percent fungal colonization) and species diversity are positively correlated with tree growth, *H2*) AMF spore production is positively correlated with tree growth, *H3*) relative abundances of specific AMF species are related to tree growth, and *H4*) AMF abundance, diversity, and spore production are correlated with chemical and biological soil characteristics, and specifically, negatively correlated with soil nitrogen (N) and P.

**Table 1:** Average tree growth (height and diameter at breast height (DBH)) and soil characteristic for all sites

Site^a^	DBH growth (cm/year)	Height growth (m/year)	Year planted	Ranked duration of pasture use^b^	pH	Organic matter (%)	C (%)	*N* (%)	P (mg/kg)	K (mg/kg)	Ca (mg/kg)	Mg (mg/kg)	Total exchange capacity (meq 100 per g)
Site 1	0.82	0.48	2004	6	5.1	15.77	6.55	.54	2	88	623	167	9.58
Site 2	0.96	0.54	2004	5	5.5	11.76	5.49	.42	3	170	1788	294	18.78
Site 3	1.17	0.53	2006	6	4.8	13.88	5.35	.55	2	55	316	70	5.7
Site 4	1.3	0.65	2005	4	5.1	17.86	6.09	.55	7	71	429	58	5.81
Site 5	1.63	1.32	2005	2	5.4	22.38	9.46	.72	2	57	1050	147	11.2
Site 6	1.81	1.35	2005	2	5.4	22.72	9.59	.79	3	68	1296	146	13.23
Site 7	1.9	1.39	2004	3	4.9	14.59	7.72	.53	2	88	802	139	12.24
Site 8	2.22	1.48	2006	1	5.6	24.81	10.93	.83	5	67	1183	185	11.54

a Site numbers are ranked based on the average DBH growth, where 1 = lowest average DBH growth and 8 = highest.

b 1 = shortest amount of time land was in pasture and 6 = longest.

## 2 Materials and Methods

### 2.1 Site description

Eight sites (50 × 50 m) distributed across a 100 km^2^ area were established between 2004 and 2006 (these eight sites were a subset of sites from Holl et al. (2011) with the highest and lowest tree growth). Sites were located near the town of Agua Buena (8° 44′ 36″ N, 82° 58′ 04″ W) and Las Cruces Biological Station (8° 47′ 7″ N, 82° 57′ 32″ W) in Coto Brus county in southern Costa Rica. This region is classified as a tropical montane rain forest (Holdridge, 1967), but due to a history of agricultural land use over the past 60 years, it has largely been deforested. Estimates show that approximately 28% (~13 km radius) is forested today, compared to 98% in the late 1940s (Zahawi, Duran, & Kormann, 2015). All sites were used for at least 18 years for agriculture and were either recently abandoned pastures dominated by exotic forage grasses or abandoned coffee farms dominated by a mixture of forage and nonforage grasses, forbs, and *Pteridium arachnoidum* (Kaulf.) Maxon (see Holl et al., 2011 for more detailed site descriptions).

The soils are classified as lixisols (ITCR 2004). In July 2012, 25 soil cores (collected at a depth of 15 cm) were taken across each site, composited, and analyzed for soil pH, organic matter (OM), percent C and N, P, cations, and micronutrients following standard procedures at Brookside Laboratories, Knoxville, OH (see http://www.blinc.com/resources/testing-methods for details on protocols).

### 2.2 Tree species

Four tree species were planted in each of the eight (50 × 50 m) sites between 2004 and 2006: two native species (*Terminalia amazonia* (J.F. Gmel.), Exell (Combretaceae) and *Vochysia guatemalensis* Donn. Sm. (Vochysiaceae)) and two naturalized, N‐fixing species (*Erythrina poeppigiana* (Walp.) Skeels and *Inga edulis* Mart. (both Fabaceae)). A total of 313 seedlings were planted in rows into each site (see Holl et al., 2011). Seedlings were acquired from a local nursery, and no mycorrhizal fungal inoculation was used at the time of planting. Height and diameter at breast height (DBH) of each tree was measured annually. Across sites, growth rates ranged from 0.8 to 2.2 cm/year for DBH and 0.5 to 1.5 m/year for height between the time of planting to 2011 (Holl et al., 2011), and the two measurements were highly correlated (*r* = .95, *p* = .0003). The eight sites were numbered based on average DBH growth (Table 1), where sites 1–4 had the lowest tree growth and sites 5–8 had the highest.

### 2.3 Mycorrhizal fungal percent colonization

To assess mycorrhizal fungal differences by site and species, ten root samples per site were randomly collected from each of the four tree species in the eight plantation sites in July 2011 (10 samples × 8 sites = 80 per tree species). Roots were examined and traced back to the adult tree to ensure that they originated from the correct tree species. The roots were subsampled for percent colonization analyses. Roots were cleared with a 10% sodium hydroxide solution and stained with a Schaeffer's ink and vinegar method (Vierheilig, Coughlan, Wyss, & Piche, 1998). Percent root length colonized was scored using a modified gridline intersections method with approximately 20 cm of root per sample (McGonigle, Miller, Evans, Fairchild, & Swan, 1990).

### 2.4 AMF species identification

Root tips from the same roots collected for the percent colonization analyses were used to identify the particular fungal species that associated with each tree species at each site. AMF DNA from approximately 25 mg of root tips were extracted with MoBio PowerSoil isolation kits (MO BIO Laboratories, Inc. Carlsbad, CA), according to the manufacturer's instructions. Root tips from ten individual trees from each site (10 trees × 8 sites = 80 per tree species) were extracted and pooled into one sample per tree species per site. The roots and DNA extracts were kept frozen or cool during transport prior to PCR amplification and sequencing. Amplification of DNA, Roche 454 sequencing, and taxonomic identification were performed by the Research and Testing Laboratory, Lubbock, TX (http://www.researchandtesting.com/).

The 18S rDNA genes in the DNA extracts, commonly used genetic markers for arbuscular mycorrhizal fungal (AMF) identification, were amplified for pyrosequencing using forward and reverse fusion primers (developed from Dumbrell et al., 2011). The fusion primers used were as follows: Forward 5′‐**GCCTCCCTCGCGCCATCAG** (10 bp MID) CAGCCGCGGTAATTCCAGCT‐3′ and Reverse 5′‐**GCCTTGCCAGCCCGCTCAG** GTTTCCCGTAAGGCGCCGAA‐3′. The forward primer was constructed (5′–3′) with the Roche A linker (in bold type), a 10‐bp barcode, and the WANDA primer (Dumbrell et al., 2011), which is a universal eukaryotic primer internal to NS31 (Simon, Lalonde, & Bruns, 1992). The reverse fusion primer was constructed (5′–3′) with the Roche B linker (in bold type) and the AM1 reverse primer (Helgason, Daniell, Husband, Fitter, & Young, 1998) which excludes plants and amplifies AMF families.

Amplifications were performed in 25 μl reactions with Qiagen HotStar Taq master mix (Qiagen Inc, Valencia, CA, USA), 1 μl of each 5 μmol/L primer, and 1 μl of template. Reactions were performed on ABI Veriti thermocyclers (Applied Biosystems, Carlsbad, CA, USA) with the following thermal profile: 95^°^C for 5 min, then 35 cycles of 94°C for 30 s, 54°C for 40 s, 72°C for 1 min, followed by one cycle of 72°C for 10 min and 4°C hold. Amplification products were visualized with eGels (Life Technologies, Grand Island, NY, USA). Products were then pooled equimolar, and each pool was cleaned and size selected using Agencourt AMPure XP (Beckman Coulter, Indianapolis, IN, USA) following Roche 454 protocols (454 Life Sciences, Branford, CT, USA). Size‐selected pools were then quantified and diluted to be used in emPCR reactions, which were performed and subsequently enriched. Samples were sequenced with a Roche 454 GS‐FLX+ system (454 Life Sciences) following established manufacture protocols.

In order to determine the identity of each sequence, sequences were clustered into operational taxonomic units (OTUs) with 100% identity (0% divergence) and compared to a database of sequences derived from NCBI (http://www.ncbi.nih.gov/) for taxonomic identification using BLASTn+ (KrakenBLAST, http://www.krakenblast.com). Sequences were then classified into the appropriate taxonomic levels based on greater than 97% sequence similarity at the species level, and 95–97% at the genus level; any match below this percent identity was discarded. In addition, the high score pair (HSP) region was at least 75% of the query sequence or it also was discarded. Nine samples failed to identify any AMF on trees roots or yield PCR product and were excluded from further analyses.

### 2.5 Spore production

Five soil samples were randomly collected in the wet and dry season (July 2011, February 2012) per site, to estimate the fungal spore community as not all AMF species sporulate at the same time (Lovelock, Andersen, & Morton, 2003). Each soil sample was a homogenized composite of three subsamples within a 1 m^2^ area. Spores were extracted from 20 g of the fresh soil matrix using multiple sieves (250, 160, and 20 μm) and the sucrose flotation method (Ianson & Allen, 1986). Sodium hexametaphosphate was used to standardize the methods and assist spore separation from the soil matrix in high clay content soils. Spores were mounted on slides using a solution of polyvinyl alcohol, lactic acid, and glycerol and then microscopically counted and identified to the genus level sensu Schenck and Pérez (1990). We were able to identify the spores only to genus level probably because a majority of the fungi associated with the roots, according to the molecular analyses, were unidentifiable or new species. Spore production was characterized by spore density and total spore volume. Spore density provides an estimate of the total number of spores produced, whereas spore volume takes in account size and germination differences and provides an estimate of the C resources used in spore production (Koske, 1987). Spore volume was calculated assuming a spherical shape and the measured diameter of each spore and then adding together the spore volumes for a particular genera or site. Estimates of spore density and volume were standardized to spores per gram of soil.

### 2.6 Data analysis

We used a mixed‐model analysis of variance (ANOVA) to analyze the relationship between tree species and AMF abundance (i.e., percent colonization, spore density, and volume) using the R project computing software (R version 3.2.3; R Development Core Team 2015). We used multiple linear regression to analyze the relationships between site‐level AMF community variables (i.e., percent colonization; fungal species diversity, richness, and evenness; spore identity, density, and volume) and site‐level tree growth (i.e., DBH). Outliers were tested with Grubbs' test for outliers (Grubbs, 1950), which is based on the assumptions of normality and compares whether the difference between the largest absolute deviation from the sample mean is larger than the sample standard deviation.

We modeled fungal species accumulation curves (R package vegan; Oksanen et al., 2013) based on individual trees and sites to determine whether we adequately sampled roots for AMF and to estimate species richness (Gotelli & Colwell, 2010). Generally, the number of individuals that must be sampled to reach an asymptote in these curves can be extremely large in the tropics (Chao, Colwell, Lin, & Gotelli, 2009), where species diversity is high and most species are rare. Therefore, we used jackknife estimators to improve accuracy and reduce bias in species richness (Palmer, 1990). We calculated species evenness to determine whether the distribution of species richness was biased (Buzas & Hayek, 2005) and used Simpson's diversity index (Simpson, 1949) due to its robustness to sample size and sensitivity to rare species (R package vegan).

Testing multiple individual soil characteristics (i.e., pH, soil OM, macronutrients, and micronutrients) for plant–soil associations would result in many comparisons and inflate type I errors. So we obtained orthogonal composite variables by computing principle components (PCs) of all soil variables (averaged at the site level) using a principal component analysis (PCA; R packages labdsv and FactoMineR; Husson, Josse, Le, & Mazet, 2015; Roberts, 2013). We chose the first three PCs, because the other PCs explained less than 10% of the variation (Table S1). We used Pearson's correlation coefficients (*r*) to characterize the relationships between ranked length of pasture use, soil variables (PCs), site‐level AMF variables (i.e., percent colonization, species diversity, spore production), and tree growth (R package Hmisc; Harrell et al., 2015).

The composition of the AMF community across tree species and sites was compared using nonmetric multidimensional scaling (NMDS; R package vegan), as it is robust to nonlinear relationships and zero inflation (Clark, 1993). NMDS was applied to a dissimilarity matrix calculated from the relative abundances of the fungal species using the Bray–Curtis dissimilarity coefficient. To test the effect of tree species identity on the AMF community composition, the community dataset was analyzed using permutational multivariate analysis of variance (PERMANOVA; Anderson, 2001), which is robust to correlations and heterogeneous variances in the dataset (Anderson & Walsh, 2013).

## 3 Results

**Figure 2:**
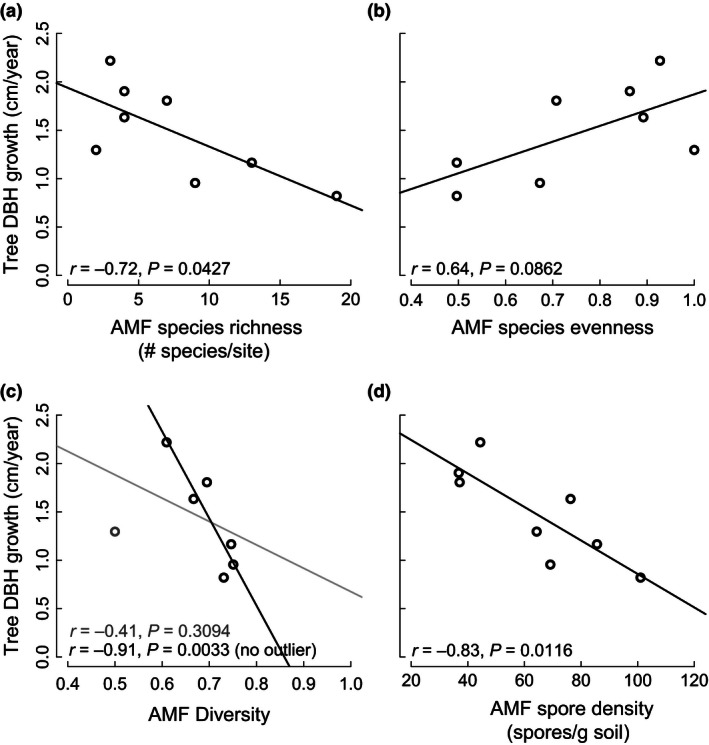
Site‐level tree diameter at breast height (DBH) growth per year as functions of (a) arbuscular mycorrhizal fungi (AMF) species richness, (b) AMF species evenness, (c) AMF diversity (gray line and text represent the diversity–DBH relationship with all data points, while the black line is without one outlier (Site 4)), and (d) AMF spore density. AMF species richness, evenness, and diversity (calculated from Simpson's diversity index) are representative of host trees' roots across all four species. AMF spore density is characterized by the number of spores per gram of soil across both wet and dry seasons

**Figure 3:**
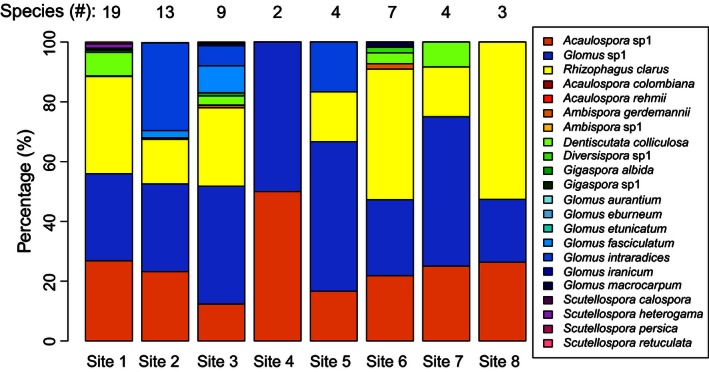
Relative abundance of arbuscular mycorrhizal fungi (AMF) within each site when omitting non‐AMF across all tree species. The number of AMF species (i.e., species richness) for each site is indicated above the respective bar

Contrary to our first hypothesis (*H1*), percent colonization was not correlated with site‐level tree growth (Table 2) but varied by site (Figure S1) and species (*F*_3, 263_ = 7.96, *p* < .0001; *E. poeppigiana*: 39.9 ± 4.2%, *I. edulis*: 48.4 ± 4.9, *T. amazonia*: 51.6 ± 3.7, *V. guatemalensis*: 53.0 ± 4.1%). Also in contrast to *H1*, the roots of low tree growth sites tended to have greater species richness (Table 2; Figure 2a) and fungal species diversity (i.e., Simpson's diversity index; Table 2 (excluding Site 4 outlier which had roots dominated by non‐AMF); Figure 2c), but marginally (α < .10) lower species evenness (Table 2; Figure 2b) than high growth sites. First‐ and second‐order jackknife estimates of species richness by the number of sites were higher than observed species richness, but there was a trend (α ≤ .10) for estimated richness differing by site‐level tree growth (*r* = −.70, *p* = .0537; *r* = −.69, *p* = .0620, respectively; see Table S2). The roots of the four tree species contained 22 AMF taxa (17 species and five identifiable only to the genus; per taxonomic classification of Schussler & Walker, 2010). Although the dominant AMF taxa differed across sites (Figure 3), three AMF taxa *(Acaulospora* sp.1, *Glomus* sp.1, and *Rhizophagus clarus*) constituted 67–100% of AMF tree associations across all sites while the other 19 taxa were rare. Over 75% of *Glomus* spp. and over 99% of *Acaulospora* spp. were unidentifiable/new species. *Gigaspora*,* Scutellospora,* and *Diversispora* spp. were only found in low growth sites and comprised less than 2% of all AMF (Table S3). At two sites (Sites 4 and 7), non‐AMF (mainly pathogenic and plant litter decomposing fungi, e.g., *Exophiala salmonis, Metacordyceps chlamydosporia, Myrothecium cinctum,* and *Volutella ciliata*) dominated the tree roots, with AMF species accounting for less than 20% of fungal inoculations.

**Table 2:** Pearson's correlation coefficients (*r*) for tree growth (i.e., DBH), ranked duration of pasture use, arbuscular mycorrhizal fungal (AMF) variables, and soil principle components (PCs) across sites. The loadings for the soil PCs from principal component analyses are in Table S1. Correlations and *p*‐values in bold type are significant (*p* < .05)

Variables	Tree growth correlation (*r*)	Tree growth*p*‐Value	Ranked duration of pasture use correlation (*r*)	Ranked duration of pasture use*p*‐Value	Soil PC2 correlation (*r*)	Soil PC2*p*‐Value
Ranked duration of pasture use	−**.92**	**.0013**	**–**	–	**–**	–
Soil PC1	.19	.653	<.01	.9947	**–**	–
Soil PC2	−**.8**	**.0173**	**.92**	**.0013**	**–**	–
Soil PC3	<.01	.997	.1	.8147	**–**	–
AMF diversity	−.41	.3094	.45	.2651	.26	.5343
AMF diversity (no outlier)	−**.92**	**.0033**	**.88**	**.0091**	**.78**	**.0394**
AMF species richness	−**.72**	**.0427**	**.77**	**.0246**	.49	.2141
AMF species evenness	.64	.0862	−**.73**	**.0381**	−.48	.2295
AMF percent colonization	.36	.3789	−.25	.5554	.04	.9167
AMF spore density	−**.83**	**.0116**	**.75**	**.0303**	.55	.1563
AMF spore volume	−.35	.3946	.24	.5748	−.02	.9641

Low growth sites had a twofold higher density of spores per gram of soil in dry (*r* = −.81, *p* = .0161) and wet (*r* = −.67, *p* = .0677) seasons than high growth sites (Figure 2d), counter to *H2*, but site‐level tree growth was not associated with the total spore volume (Table 2). Spore density did not differ between seasons (*F*_1,13_ = 1.97, *p* = .1845), and dry season spore volume was marginally (α ≤ .10) greater than wet season (*F*_1,13_ = 3.17, *p* = .0985). Similar to the root results, the majority of the spores by number and volume were members of the genus *Glomus* (95%; 61%, respectively) and genus *Acaulospora* (5%; 32%, respectively). *Glomus* had higher spore density in low growth sites, regardless of season (*r* = −.84, *p* = .0101), while *Gigaspora*, in the dry season, had marginally higher spore density (*r* = −.62, *p* = .1054) and volume (*r* = −.69, *p* = .0552) in low growth sites but was <1% of overall spore numbers and volume.

Inconsistent with our expectation (*H3*), tree growth was not correlated with the relative abundance of specific AMF taxa (*I. edulis*:* F*_1,7_ = 0.68, *p* = .6078; *E. poeppigiana*:* F*_1,7_ = 0.82, *p* = .5341; *T. amazonia*:* F*_1,7_ = 0.58, *p* = .6856; *V. guatemalensis*:* F*_1,7_ = 0.22, *p* = .9146). The relative abundances of the AMF community also were not influenced by host tree identity (Figure S2), and particular fungi did not associate with only one tree species. *V. guatemalensis* associated with the most AMF taxa (17), followed by *E. poeppigiana* (15)*, I. edulis* (14)*,* and *T. amazonia* (12). First‐ and second‐order jackknife estimates of species richness by individual trees were slightly higher than actual species richness but similar across tree species (see Table S2). In *T. amazonia* and *I. edulis*, fungal taxa from the genus *Glomus* dominated (58 and 57% of AMF, respectively), whereas *R. clarus* comprised 49% of *V. guatemalensis* fungal‐tree symbioses. A combination of *Glomus* and *Acaulospora* made up 33 and 27%, respectively, of symbioses with *E. poeppigiana*.

Arbuscular mycorrhizal fungal species diversity, but not spore density or volume, was negatively correlated with OM, C, and N concentrations (*H4*; PC 2 in Table S1; Table 2). Soils were acidic (pH ~5.2), had a clay‐enriched subsoil, low to moderate levels of exchangeable cations (calcium, magnesium, and potassium), and low P levels; soil OM also was high (>10%; Table 1). Lower OM, C, and N concentrations (PC 2) also were correlated with higher site‐level fungal colonization on only *V. guatemalensis* roots (*r* = −.73, *p* = .0389).

Additionally, sites with longer duration of previous pasture use had higher AMF species diversity (without outlier), higher species richness, higher spore density, lower soil OM, C, and N concentrations (PC 2), and lesser species evenness, but pasture duration was not related to fungal colonization or spore volume (Table 2). Longer duration of pasture use and greater AMF diversity was similarly strongly correlated with tree growth, whereas higher spore densities and lower soil OM, C, and N were less strongly correlated with tree growth (Table 2).

## 4 Discussion

Contrary to our hypotheses (*H1* and *H2*), tree growth was negatively correlated with greater AMF species richness, diversity, and spore density in restored pastures (Figure 2). AMF richness, diversity, and spore density also positively correlated with longer duration of pasture use and could explain some influence of prior land use on tree growth (Holl & Zahawi, 2014). The majority of fungal species and spores belonged to *Glomus* and *Acaulospora,* consistent with their ability to tolerate soil disturbances (Boddington & Dodd, 2000) and similar to prior tropical restoration research (Allen, Allen, Egerton‐Warburton, Corkidi, & Gomez‐Pompa, 2003; Haug et al., 2010). Conversely, negative correlations between AMF diversity and soil OM, C, and N (*H4*) was in accordance with previous research (Camenzind et al., 2014; Egerton‐Warburton & Allen, 2000; Lekberg et al., 2007) as well as soil relationships with pasture use and tree growth (see Guariguata & Ostertag, 2001). For this study, we cannot distinguish whether soil attributes (OM, C, and N) or mycorrhizal diversity directly influenced tree growth, but there could be a role for both.

The negative AMF–plant growth relationship may have arisen from soil attributes causing variation in both tree growth and AMF diversity. Trees can facultatively increase their associations with fungal symbionts under degraded environmental conditions (Johnson, Graham, & Smith, 1997; Smith, Grace, & Smith, 2009), such that AMF richness (Egerton‐Warburton & Allen, 2000), AMF abundance (Treseder, 2004), and plant C allocation to AMF structures (Johnson, Rowland, Corkidi, Egerton‐Warburton, & Allen, 2003) may increase with decrease in soil fertility. Consistent with our prediction (*H4*), AMF diversity was negatively correlated with soil characteristics (i.e., OM, C, and N), but AMF abundance or spore production did not vary (Table 2). Higher levels of soil OM, C, and N also strongly associated with greater tree growth and shorter pasture use, even though soil attributes in previous studies (i.e., Holl & Zahawi, 2014; Holl et al., 2011) explained very little of the variation in tree growth potentially due to differences in data analysis (PCA composites vs. regression with Bonferroni's corrections). The more diverse AMF communities at low growth and soil fertility sites could indicate those trees' greater need for fungal symbionts. But as AMF diversity increased at low growth sites, the overall amount of fungal root colonization did not vary with site‐level tree growth (*H1*), which is the characteristic measurement for plant allocation to fungal structures. Although changes in root colonization are typically interpreted as alterations of host plant C allocation to fungi, empirical evidence is weak and percent colonization is most likely determined by both plants and fungi (Kiers et al., 2011; Maherali & Klironomos, 2007). Thus, we propose that longer pasture use reduced soil fertility, which in turn may have increased dependence on AMF and resulted in greater AMF richness and diversity in sites with lower tree growth.

Differences in AMF community composition across sites may be a result of soil fertility. In this study, *Glomus* and *Acaulospora* were the dominant AMF taxa across all sites, which can have different niche space on roots (Maherali & Klironomos, 2007, 2012). However, only our low growth sites (i.e., sites with longer durations of pasture use and less soil OM, C, and N) had *Gigaspora* and *Scutellospora* colonization and greater densities of *Glomus* spores. Nitrogen enrichment can facilitate the displacement of *Gigaspora* and *Scutellospora* with the proliferation of *Glomus* (Egerton‐Warburton & Allen, 2000); *Glomus* are thought to be better adapted to disturbed environments due to their high sporulation rates (Daniell, Husband, Fitter, & Young, 2001) and their ability to rapidly colonize via fragments of mycelium or mycorrhizal roots (Biermann & Linderman, 1983). Soil OM also can act as a nutrient source (Jayachandran, Schwab, & Hetrick, 1992; Thirkell, Cameron, & Hodge, 2016) and influence AMF composition; in soils with low organic content (i.e., our low growth sites), *Gigaspora* and *Scutellospora* can predominate, whereas *Glomus* tend to be more abundant in high organic soils (Lekberg et al., 2007). In addition, extensive extraradical hyphal growth in *Gigaspora* and *Scutellospora* (Hart & Reader, 2002a) can increase nutrient acquisition compared to *Glomus* and *Acaulospora* (Maherali & Klironomos, 2007, 2012), thus potentially favoring *Gigaspora* and *Scutellospora* in our lower fertility soils. Although we are unable to untangle the effects of soil characteristics versus AMF diversity on tree growth in this study, the distribution of specific AMF taxa across sites could have influenced the AMF–tree relationship.

The observed negative relationship between AMF and plant growth, on the other hand, also may be a result of trees in low growth sites associating with multiple, inefficient fungi. The destruction of soil structure, specifically from disturbances such as longer pasture duration in our low growth sites, can promote the proliferation of less mutualistic fungi (Bever et al., 2009). As not all AMF species are equally beneficial to host plants, C allocated to multiple, less efficient fungi could result in reduced plant growth (Kiers et al., 2011). Although plants may preferentially allocate C to more beneficial AMF (Kiers et al., 2011), they may not strongly control the initial stages of AMF colonization (Akiyama, Matsuzaki, & Hayashi, 2005; David‐Schwartz et al., 2003), so that less efficient fungi can colonize roots before more beneficial AMF. Thus, due to the correlative nature of this study, we cannot determine whether AMF overall improved and/or hindered tree growth.

### 4.1 Methodological considerations

Although more individual species were found in tree roots at low growth sites (Figure 2a), there were a few common species and many rare ones, as evidenced from species evenness measurements (Figure 2b) and the narrow range of diversity indices (Figure 3c) across sites. These uneven species abundances and the lack of correlation between AMF root colonization and tree growth could have been a consequence of measuring only fungal structures internal to the trees' roots (obtained by fungal DNA isolation from root fragments and measuring the percent of internal root length colonized by fungi), which also may have underestimated the presence, abundance, and thus influence of rare species. Whereas the diffuse internal hyphae and sparse external structures of *Glomus* and *Acaulospora* would be adequately represented by our relative abundance measurements, *Gigaspora* tends to produce densely aggregated internal hyphae and long external hyphae which would be poorly represented by internal root measurements (Hart & Reader, 2002b). Thus, our many “rare” species could be underestimated and may be more “common” than represented in this study. Whereas we have good estimates of AMF colonization and internal species abundances, we acknowledge that this study is lacking a complete picture of AMF biomass. These limitations, combined with many fungal individuals unidentifiable to species level, constrain our ability to fully evaluate the relationships among tree growth and AMF abundance (*H1*), AMF diversity (*H1*), and specific AMF taxa (*H3*).

While the primer pair NS31/AM1 used in our study is common in field‐based studies of AMF communities from various geographic locations and ecosystems (e.g., Hazard et al., 2013; Husband, Herre, Turner, Gallery, & Young, 2002; Opik, Moora, Liira, & Zobel, 2006), the AM1 primer only amplifies the central fragment of the 18S rDNA gene (Helgason et al., 1998) and excludes many species within the Archaeosporaceae and Paraglomeraceae families (Daniell et al., 2001; Lee, Lee, & Young, 2008; but also see exceptions in Helgason, Fitter, & Young, 1999; Wirsel, 2004). Whereas most of Glomeromycota's natural diversity is found in this gene region, it is possible that the primers used in this study biased our AMF diversity values, resulting in three taxa containing the majority of the observed mycorrhizal fungi across all sites. However, Dumbrell et al. (2011) argue that using more inclusive primer sets could still result in low numbers from the “excluded” families (e.g., Lumini, Orgiazzi, Borriello, Bonfante, & Bianciotto, 2010; Santos‐Gonzalez, Finlay, & Tehler, 2007), suggesting that these taxa may be relatively rare and that using the NS31/AM1 primers do not substantially underestimate AMF diversity.

Greater AMF diversity in low growth sites may have been a consequence of greater root sampling. Although aboveground tree growth was lower in low growth sites, we cannot determine whether root biomass differed among sites as it was not measured. Lower aboveground growth could suggest smaller biomass belowground, but it also might indicate greater resource allocation to roots. As all trees roots were similarly subsampled, we may have sampled more roots from low growth sites if those sites also had lower root biomass.

We also did not measure the fungal composition of sites prior to tree planting. Although higher AMF diversity and spore density were associated with reduced tree growth 5–7 years after transplanting, we do not know whether the AMF community changed during that time. Land use changes (i.e., forest to pasture) can alter the taxonomic composition of AMF communities (Aldrich‐Wolfe, 2007) but not necessarily species richness and abundance (Leal, Siqueira, & Sturmer, 2013).

## 5 Conclusions

Although numerous studies have found a positive link between the AMF community and plant growth, our negative plant growth–fungal diversity relationship indicates that multiple factors may be influencing tropical restoration efforts. While AMF richness, diversity, and spore density may explain some of the influence of prior land use on tree growth, strong associations between soil attributes (OM, C, and N) and AMF diversity suggest a facilitative relationship between trees and their fungal symbionts. Changes to AMF community composition also may lead to differences in nutrient acquisition and susceptibility to disturbances, which could influence AMF–tree relationships. Even though we cannot untangle the effects of soil attributes and mycorrhizal fungal community on tree growth, this research highlights the importance of considering mycorrhizal symbionts in the growth of tropical trees, especially in a restoration context.

## Conflict of Interest

None declared.
