# Supplementary material for: Reduced aboveground tree growth associated with higher arbuscular mycorrhizal fungal diversity in tropical forest restoration
Source: Ecol Evol. 2016 Sep 21;6(20):7253–62. doi: 10.1002/ece3.2487 (PMC5513279; doi:10.1002/ece3.2487)
Supplement: Supplementary file 1 [file ECE3-6-7253-s001.docx]

**Table S1.** Soil variable loadings for the eight sites from Principal Component Analyses (PCA). Variance values indicate the percentage of the total variance accounted for by each principal component (PC). Other PCs explained less than 10% of the variation. Individuals in bold type were the significant variables (P<0.05) in each PC.

| Soil Variables | PC 1 | PC 2 | PC 3 |
| --- | --- | --- | --- |
| pH | -0.236 | -0.293 | -0.127 |
| Organic Matter | --- | **-0.405** | --- |
| C:N | -0.205 | -0.159 | --- |
| C | --- | **-0.418** | --- |
| N | 0.101 | **-0.405** | --- |
| P | --- | --- | **-0.639** |
| K | **-0.337** | 0.173 | --- |
| Ca | **-0.335** | -0.167 | --- |
| Mg | **-0.369** | --- | --- |
| Total Exchange Capacity | **-0.353** | --- | --- |
| S | **0.297** | --- | -0.412 |
| Na | 0.171 | 0.265 | 0.201 |
| Fe | -0.180 | **0.346** | --- |
| Mn | -0.165 | 0.293 | -0.327 |
| Cu | --- | -0.151 | -0.386 |
| Zn | **-0.307** | --- | 0.115 |
| Al | **0.342** | --- | 0.224 |
| Variance (%) | 41 | 32 | 11 |

**Table S2.** Estimates of species richness for each tree species and site based on species accumulation curves.

| Variable | Observed species richness | First-order jackknife species richness estimate  ± standard error | Second-order jackknife species richness estimate |
| --- | --- | --- | --- |
| Tree species  *E. poeppigiana* | 15 | 23.8 ± 7.6 | 30.0 |
| *I. edulis* | 14 | 19.3 ± 4.1 | 21.8 |
| *T.* amazonia | 12 | 19.0 ± 7.4 | 23.7 |
| *V. guatemalensis* | 17 | 24.9 ± 6.6 | 30.3 |
| Sites  Site 1 | 19 | 26.5 ± 6.1 | 30.2 |
| Site 2 | 10 | 13.8 ± 4.2 | 15.9 |
| Site 3 | 13 | 14.5 ± 1.1 | 14.2 |
| Site 4 | 3 | 4.5 ± 1.1 | 5.2 |
| Site 5 | 5 | 8.0 ± 2.6 | 9.7 |
| Site 6 | 8 | 12.5 ± 5.1 | 14.8 |
| Site 7 | 5 | 7.3 ± 1.8 | 8.4 |
| Site 8 | 4 | 5.5 ± 1.6 | 5.8 |

**Table S3.** Relative abundance of each arbuscular mycorrhizal fungi (AMF) within each site when omitting non-AMF. Individuals in bold type only were identifiable to the genus level.

| Genera | AMF Species | Site 1 | Site 2 | Site 3 | Site 4 | Site 5 | Site 6 | Site 7 | Site 8 |
| --- | --- | --- | --- | --- | --- | --- | --- | --- | --- |
| *Acaulospora* | ***Acaulospora* sp1** | 26.82 | 23.17 | 12.31 | 50.00 | 16.67 | 21.82 | 25.00 | 26.32 |
| *Acaulospora* | *Acaulospora colombiana* | -- | 0.27 | -- | -- | -- | -- | -- | -- |
| *Acaulospora* | *Acaulospora rehmii* | -- | 0.41 | -- | -- | -- | -- | -- | -- |
| *Ambispora* | ***Ambispora* sp1** | 0.05 | -- | 0.15 | -- | -- | -- | -- | -- |
| *Ambispora* | *Ambispora gerdemannii* | 0.12 | -- | 0.76 | -- | -- | 1.82 | -- | -- |
| *Dentiscutata* | *Dentiscutata colliculosa* | 7.88 | 0.07 | 3.04 | -- | -- | 3.64 | 8.33 | -- |
| *Diversispora* | ***Diversispora* sp1** | 0.09 | -- | -- | -- | -- | 1.82 | -- | -- |
| *Gigaspora* | ***Gigaspora* sp1** | 0.01 | -- | -- | -- | -- | -- | -- | -- |
| *Gigaspora* | *Gigaspora albida* | 0.61 | -- | 1.04 | -- | -- | -- | -- | -- |
| *Glomus* | ***Glomus* sp1** | 29.16 | 29.37 | 39.43 | 50.00 | 50.00 | 25.45 | 50.00 | 21.05 |
| *Glomus* | *Glomus aurantium* | 0.05 | -- | -- | -- | -- | -- | -- | -- |
| *Glomus* | *Glomus eburneum* | 0.04 | -- | -- | -- | -- | -- | -- | -- |
| *Glomus* | *Glomus etunicatum* | 0.03 | -- | -- | -- | -- | -- | -- | -- |
| *Glomus* | *Glomus fasciculatum* | -- | 2.39 | 9.03 | -- | -- | -- | -- | -- |
| *Glomus* | *Glomus intraradices* | 0.40 | 29.37 | 6.73 | -- | 16.67 | -- | -- | -- |
| *Glomus* | *Glomus iranicum* | 0.01 | -- | 0.02 | -- | -- | -- | -- | -- |
| *Glomus* | *Glomus macrocarpum* | 0.02 | 0.03 | 0.75 | -- | -- | 1.82 | -- | -- |
| *Rhizophagus* | *Rhizophagus clarus* | 32.51 | 14.92 | 26.28 | -- | 16.67 | 43.64 | 16.67 | 52.63 |
| *Scutellospora* | *Scutellospora calospora* | 0.15 | -- | -- | -- | -- | -- | -- | -- |
| *Scutellospora* | *Scutellospora heterogama* | 1.44 | -- | 0.30 | -- | -- | -- | -- | -- |
| *Scutellospora* | *Scutellospora persica* | 0.01 | -- | -- | -- | -- | -- | -- | -- |
| *Scutellospora* | *Scutellospora reticulata* | 0.57 | -- | 0.15 | -- | -- | -- | -- | -- |


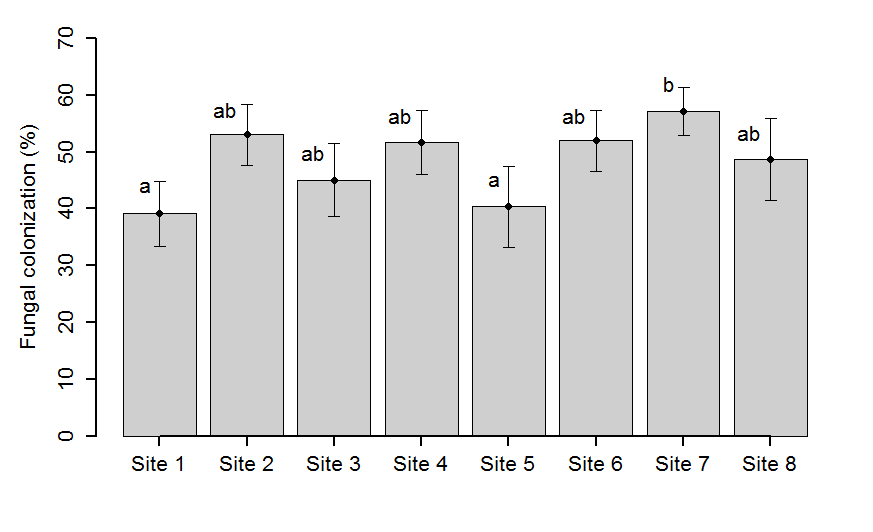


**Figure S1.** Percent arbuscular mycorrhizal fungal (AMF) colonization for each site across all four tree species (*E. poeppigiana, I. edulis, T. amazonia,* and *V. guatemalensis*). Bars represent standard errors, and letters denote significant differences (P<0.05) between sites.


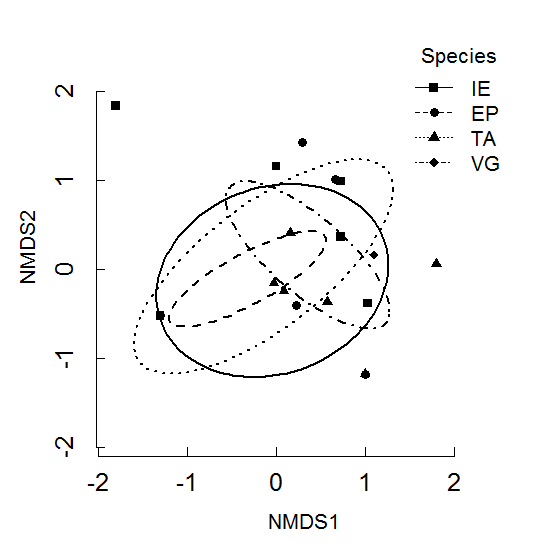


**Figure S2.** Non-metric dimensional scaling plot (2 Dimensional NMDS, Stress=0.07, R^2^=0.995) of the arbuscular mycorrhizal fungal (AMF) community structure classified by tree species identity (IE=*I. edulis*, EP= *E. poeppigiana*, TA=*T.* amazonia, VG=*V.guatemalensis*). Each point represents a composite of the AMF community of a given tree species per site, and ellipses show the overlap in the fungal community across tree species (confidence area of ellipses = 0.95). PERMANOVA tests showed no differences in the AMF community between tree species (F_3,22_=1.19, P=0.3003).
